# Low temperatures and wind; challenges, applicability and limitations from an industrial perspective

**DOI:** 10.1186/2046-7648-4-S1-A162

**Published:** 2015-09-14

**Authors:** Øystein Nordrum Wiggen, Arild Øvrum, Arne Haugan, Hilde Færevik

**Affiliations:** 1 SINTEF Technology and Society, Department of Health Research, Trondheim, Norway; 2 Statoil ASA

## Introduction

The effect of low temperature and wind on the cooling of exposed skin has been a field of interest since the first study by Siple and Passel in 1945[1]. This resulted in the Wind Chill Index (WCI), which expressed the rate of heat loss of a cylinder per unit surface mass. The WCI remained unchanged for many years, and is still regarded as an established and well-defined framework for assessing cold stress. However, in recent years, the Wind Chill Equivalent Temperature (WCT) has supplanted the WCI. WCT is a calculated air temperature based on dry-bulb temperature and wind speed. In the last number of years, these equations have undergone several revisions, in an attempt to develop a tool that is easy to understand and use, and is scientifically valid. The rise in industrial activity in the High North has brought new challenges and a need for better winterisation standards in installations and vessels designed to operate in extremely cold climates. Reducing the need for winterisation without compromising the health and safety of the workers will reduce both building costs and the weight of offshore installations. The aim of this study was to compare the wind-chill recommendations in ISO 11079 and NORSOK S-002, Working Environment.

## Methods

The existing ISO 11079 [2] and NORSOK S-002 [3], Working Environment were compared, and a narrative review of the relevant scientific background of the different wind chill indices and equivalent temperatures were performed.

## Results

The NORSOK S-002, Working Environment refers to ISO 11079, but presents WCI contrary to ISO 11079 that presents WCT. The critical threshold values are therefore different between the two standards.

## Discussion

The WCT is first and foremost a tool for ensuring the health and safety of personnel who work in cold and windy environments. For the safe and efficient design and operation of installations in such environments, a common understanding of how to apply the WCT is essential. In the design of both on- and offshore installations, the older WCI is still used to define the threshold values applied to cold working conditions. Engineers prefer the use of WCI (W/m^-2^) in their calculations; however, due to the differences in the weighting of wind and temperature between WCI and WCT, it is not possible to use both measures. The WCI exaggerates the effect of wind compared to WCT. A low air temperature is needed for the skin to freeze, while a two-fold increase in temperature doubles the heat flow rate, whereas doubling wind speed only increases the heat flow rate by 50% [4]. WCI results in a more conservative approach being taken to cold working conditions and potentially to unnecessarily stringent requirements for winterisation.

## Conclusions

The WCT chart in current use is employed as a uniform cold stress standard, perhaps for a broader audience than was initially intended. Challenges still remain regarding the use of WCT in the whole process from design to workplace monitoring.
